# Oncologists’ Locus of Control, Compassion Fatigue, Compassion Satisfaction, and the Mediating Role of Helplessness

**DOI:** 10.3390/curroncol29030137

**Published:** 2022-03-04

**Authors:** Michal Braun, Lee Naor, Ilanit Hasson-Ohayon, Gil Goldzweig

**Affiliations:** 1 Oncology Breast Unit, Sharett Institute of Oncology, Hadassah Medical Center, Jerusalem 9574401, Israel; 2 School of Behavioral Sciences, The Academic College of Tel Aviv-Yaffo, Yaffo 6818211, Israel; leenaormail@gmail.com (L.N.); giligold@mta.ac.il (G.G.); 3 Psychology Department, Bar-Ilan University, Ramat Gan 5290002, Israel; hasoni@mail.biu.ac.il

**Keywords:** oncology, cancer, compassion fatigue, compassion satisfaction, locus of control, guilt, helplessness

## Abstract

The oncology setting may give rise to significant feelings of helplessness among oncologists via patients’ inevitable deaths or suffering. The current study examines whether and how oncologists’ sense of control (locus of control; LOC) influences their compassion fatigue and satisfaction. **Methods:** Seventy-three oncologists completed the following questionnaires: the Professional Quality of Life scale; Levenson’s Internal, Powerful Others, and Chance scale; the Guilt Inventory, State Guilt subscale; and the Learned Helplessness scale. **Results:** Oncologists reported high levels of secondary traumatic stress and burnout and moderate levels of compassion satisfaction. A positive association between oncologists’ external LOC and compassion fatigue, and a negative association between oncologists’ internal LOC and compassion fatigue, were found. Helplessness, but not guilt, had a mediating role in these associations. Internal LOC was also positively associated with compassion satisfaction. **Conclusions:** The current study highlights oncologists as a population at risk of experiencing compassion fatigue and emphasizes oncologists’ locus of control as a predisposition that plays a role in the development of this phenomenon. Additionally, the cognitive as well as the emotional aspects of control were found to be important factors associated with compassion fatigue.

## 1. Introduction

In 2020, the American Society of Clinical Oncology (ASCO) published a call to action regarding burnout, compassion fatigue, moral distress, and negative well-being among oncologists [1]. This call to action was published on the basis of growing interest in and evidence of the potential price of working as a healthcare professional. In the last decade, it has been established that burnout is a widespread, central, and important problem faced by the medical world [2,3]. For example, it was found in an extensive review that physicians’ burnout prevalence—evidence of which was extracted from 182 studies involving 109,628 individuals in 45 countries, and published between 1991 and 2018—was 67% [4].

Oncology is a medical subspecialty that requires special consideration in terms of burnout and compassion fatigue, given the physical and mental suffering and death and dying of patients that healthcare providers are exposed to daily, as well as the long-term and extensive oncologist–patient relationship. This extensive exposure along with the goal of alleviating suffering and prolonging patients’ lives might lead healthcare providers to experience the abovementioned phenomena (i.e., compassion fatigue and burnout). Indeed, burnout and compassion fatigue occur frequently among oncology clinicians [5,6,7,8,9,10,11,12,13,14,15]. For example, in a survey of ASCO member medical oncologists, 45% reported experiencing emotional exhaustion and/or depersonalization related to burnout [5]. In a Hunt et al. study, a quarter of the oncology caregivers reported high levels of compassion fatigue [6]. Furthermore, two systematic reviews and meta-analyses reported that a significant number of oncologists experience high levels of burnout [12,13], and we reported high levels of compassion fatigue (secondary traumatic stress/STS and burnout) among oncologists in a previously published paper based on the current sample [14]. Burnout and compassion fatigue may affect oncology clinicians’ physical and mental health, their decision-making ability, their committing of medical errors, their relationships with patients and families, and the quality of care they provide [7,13]. However, research in oncology is limited, especially among oncologists. That said, there is some evidence that oncologists report even higher levels of burnout compared to oncology nurses [12]. The ASCO call to action stated that more studies are needed to clarify the causes and associations between burnout, moral distress, and compassion fatigue.

There is confusion in the literature regarding the different concepts used to describe the negative consequences of working as a health care provider, such as compassion fatigue, burnout, secondary traumatic stress, and vicarious trauma. In the current study, we used Stamm’s model. Stamm [16,17] developed a comprehensive theoretical model that conceptualizes compassion fatigue as the negative aspects of working in the helping professions, and compassion satisfaction as the positive aspects. Compassion fatigue is defined as a state of tension, which can cumulatively develop in the helping professions in response to witnessing others’ suffering as well as the constant wish to relieve this suffering. The consequences of compassion fatigue include the caregiver’s reduced capacity and interest in being empathetic towards others who are suffering, and in reduced personal and professional well-being. In Stamm’s model [16,17] compassion fatigue is composed of burnout and secondary traumatic stress, both of which are regarded as negative implications of the work. Burnout usually develops in response to work environment characteristics (e.g., workload and non-supportive work environment) and includes feelings of exhaustion, frustration, anger, and depression. Secondary traumatic stress develops in response to exposure to patients’ traumatic events, and its symptoms include intrusive thoughts, avoidant behavior, and high levels of tension. By contrast, compassion satisfaction is regarded as the positive feelings that professionals might derive from practicing their field of medicine, such as the pleasure of helping others and the feeling of success in helping others. It is important to note that compassion satisfaction and compassion fatigue exist independently from one another, and can also co-exist, as can be seen in the ASCO survey; specifically, almost half of the oncologists reported symptoms of burnout, but at the same time more than 80% of them were satisfied with their career and choice of specialty [5]. In Granek’s study, oncologists reported high levels of compassion fatigue, while at the same time reporting high levels of secondary traumatic stress [11].

Much of the research thus far on this topic focuses on documenting the prevalence of burnout and compassion fatigue, but there is limited knowledge about the factors that may contribute to them. In order to understand and guide future interventions better, it is important to focus on the emotional and psychological constructs that explain these phenomena. In the current study, we examined whether and how oncologists’ sense of control influenced their compassion fatigue and satisfaction. The oncology setting puts medical staff in the position of having significant encounters with feelings of helplessness, including being unable to avoid patients’ inevitable deaths or having difficulty in alleviating patients’ suffering. As such, oncologists’ sense of control might be an important resource in their ability to cope with these kinds of challenges. Locus of control (LOC), according to Rotter [18], can be defined as the way individuals attribute causes to the occurrence of various events in their lives. Individuals with an internal LOC perceive events as dependent on the efforts they exert to make them happen: That is, they believe that their own abilities, efforts, and actions influence reality. In contrast, individuals with an external LOC do not see an association between their efforts and the way things happen in reality. They believe that fate, luck, or external forces are responsible for the occurrence of events. Levenson [19] further divided external LOC into two separate components. The first is “powerful others”: seeing others (people or God) as having control over events. The second is “chance”: the belief that events are affected by luck and chance only.

Internal LOC has frequently been found to be associated with favorable outcomes. For example, in a very large meta-analysis (222 papers over 20 years) on LOC in the workplace, internal LOC was found to be positively associated with (among other things) mental well-being, life satisfaction, job satisfaction, task performance, and career success, and negatively associated with (among other things) job stress and burnout [20]. The same pattern was found in the only study we found that examined oncologists’ LOC. Ramondetta et al. found that higher levels of external LOC were positively associated with work-related stress, whereas internal LOC was negatively associated with it [21]. That said, the oncology setting differs from many other settings in that healthcare workers who work in this setting are essentially required on a daily basis to face situations in which the reality is uncontrollable. In their daily work, oncologists must repeatedly face their own inability to change the course of the disease or even to alleviate patients’ suffering. Therefore, we wished to look at oncologists’ LOC and its association with compassion fatigue and compassion satisfaction. Locus of control is a cognitive structure representing perceptions and interpretations of events, but we also examined possible emotional mediating variables—namely, guilt, and helplessness. The study thus offers these two variables as potential explanatory factors for the associations between LOC and oncologists’ compassion fatigue. It is important to note that although both guilt and helplessness are negative emotions, guilt seems intuitively to be associated with internal LOC and helplessness with external LOC.

Guilt is defined as an unpleasant feeling accompanied by the belief that one should have thought, felt, or acted differently. Although it plays an important role in interpersonal relationships, it also exerts a strong effect on one’s personal well-being [22]. Specifically, oncologists reported feeling guilt in several studies [23,24,25] and it was found to influence both oncologists’ personal well-being as well as their professional performance [23]—for example, their decision-making processes [25]. Guilt among oncologists may develop as a result of the intersection between treatment failure, an inability to alleviate patients’ suffering, and patients’ deaths [23,24,25] and their sense of responsibility and excessive, self-imposed requirements [23,24]. Nowakowski et al. [24] called this phenomenon unjustified guilt and found that 81% of the oncologists in their study experienced at least one episode of it.

It seems that in order to experience guilt, individuals must attribute the event in question to their own failures and, therefore, guilt might mediate a possible positive association between internal LOC and compassion fatigue. As such, we assumed that a possible positive association might be found between internal LOC and compassion fatigue due to the unique characteristics of the oncology setting, namely, the guilt that might be aroused in light of the encounter between the reality and oncologists’ tendency to attribute the reality to their own efforts and actions might result in compassion fatigue.

In this study, we also examined another mediating model in which learned helplessness might serve as a mediator in the associations between LOC and compassion fatigue and between LOC and compassion satisfaction. Learned helplessness is a psychological condition, which develops as a result of cumulative exposure to uncontrollable experiences and individuals’ perceptions that their actions have no influence on events [26]. Rotter [18] suggested that when individuals hold external LOC attributions, these external LOC attributions can be manifested in (among other things) learned helplessness. Stamm [16,17] emphasized that one of the most prominent symptoms of compassion fatigue is the feeling of despair and hopelessness. Such feelings might be affected by an external LOC and helplessness. Oncologists who have an external LOC, who believe that external forces are responsible for their patients’ health and well-being, may develop learned helplessness, which in turn may lead to the development of compassion fatigue. In contrast, oncologists who have an internal LOC, who believe that they have an influence on their patients’ health and well-being, might feel less helpless and therefore suffer to a lesser degree from compassion fatigue.

The current study was conducted in order to understand those psychological characteristics and emotional factors of oncologists that might contribute to compassion fatigue and compassion satisfaction better. As such, we examined the association between oncologists’ LOC and compassion fatigue (i.e., comprising both burnout and secondary traumatic stress) and between oncologists’ LOC and compassion satisfaction, and suggested two possible mediating variables: guilt and helplessness. It is important to note that although we were able to pose hypotheses regarding the possible associations with compassion fatigue, due to the void in the theoretical and empirical literature, we did not pose hypotheses regarding compassion satisfaction; rather, we only explored the associations between guilt, helplessness, and compassion satisfaction.

## 2. Method

The current study is part of a large-scale study on oncologists’ compassion fatigue and satisfaction. Whereas previous studies from this project focused on the role of grief and sense of failure in the experience of compassion fatigue [14] and the mediating role of compassion fatigue in the association between empathy and grief [27], the current study focused on oncologists’ LOC and guilt and helplessness as possible mediating variables.

### 2.1. Participants and Procedure

The current study participants were Israeli oncologists recruited at the 2018 Spring Meeting of the Israel Society of Hematology and Blood Transfusion, and the 2018 Annual Meeting of the Israeli Society for Clinical Oncology and Radiation Therapy. The study was presented to the oncologists through brochures that were distributed at the meetings and face to face. They entered the study’s questionnaires through a link that was introduced to them in the brochures. A total of 92 of the oncologists consented to participate in the study, and 73 provided complete data. The participants signed informed consent and filled out the questionnaires via Qualitrics^®^ during the meetings. A lottery ticket was offered as an incentive to participate. This study received approval from the institutional review board of the Academic College of Tel Aviv–Yaffo (approval No. 2018001).

### 2.2. Measures

The formal Hebrew language version of the **Professional Quality of Life scale, ProQOL** (Version 5) [17], was used to assess compassion satisfaction and the two components of compassion fatigue: secondary traumatic stress and burnout. This measure consists of 30 items, with a 6-point response range scale for each item. The questionnaire includes 3 subscales (10 items each): secondary traumatic stress (e.g., “I feel depressed because of the traumatic experiences of the people I help”), burnout (e.g., “I feel trapped by my job as a helper”), and compassion satisfaction (e.g., “I get satisfaction from being able to help people”). In the present study, the Cronbach alpha coefficients were adequate (secondary traumatic stress: α = 0.79, burnout: α = 0.72, compassion satisfaction: α = 0.87).

**Levenson’s Internal, Powerful Others, and Chance (IPC) scale** [19], Hebrew version [28], was used to assess the LOC. The questionnaire distinguishes between three types of LOC: internal LOC (e.g., “My ability is what determines whether I will be an influential person”), external LOC/powerful others (e.g., “I feel that what happens in my life is determined by the people who have a lot of influence on me”), and external LOC/chance (e.g., “Life is managed by random events”). This measure consists of 24 statements (8 statements per subscale) rated on a Likert scale ranging from 0–4. The score for each subscale is based on the sum of items, and ranges from 0 to 32. In this study, Cronbach’s alpha for the internal LOC was high (0.80), and for external LOC/powerful others and external LOC/chance, it was moderate (0.76; 0.70).

**The Guilt Inventory, State Guilt subscale** [29], Hebrew version, was used to assess guilt. This subscale includes 10 statements that measure situational guilt—that is, feelings of guilt attributed to the present period (e.g., “I recently did something I very much regret”). Each item is rated on a Likert scale ranging from 1 to 5. The average of the items is calculated, with higher numbers reflecting a greater sense of guilt. Cronbach’s alpha in this study was high, 0.87.

**The Learned Helplessness scale** [30], Hebrew version [31], was used to assess helplessness. The questionnaire includes 20 statements (e.g., “I have a feeling nothing will help me”). Each item is rated on a Likert scale ranging from 1 to 4. The average of the items is calculated, with higher numbers reflecting a higher degree of helplessness. In the current study, Cronbach’s alpha was high, 0.96.

**A demographic questionnaire** consisted of questions regarding personal information (e.g., age and gender) and professional information (e.g., specialty and place of work).

### 2.3. Statistical Analysis

Data were analyzed using the Statistical Package for the Social Sciences (SPSS), version 25.0. Descriptive statistics were calculated for the sociodemographic variables and for the main study variables: compassion fatigue, compassion satisfaction, LOC, guilt, and helplessness. To test the study’s hypothesis—namely, that oncologists’ guilt and helplessness would mediate the effects of oncologists’ LOC on their compassion fatigue and satisfaction—structural equation modeling (SEM) was applied with SPSS AMOS version 25.

## 3. Results

Data from 73 oncologists were included in the analysis. The participants’ mean age was 44.75 years (SD = 11.37, range: 29 to 75); 42 oncologists were female (57.5%); 70 (95.9%) were married or living with a partner; and 50 (68.5%) were born in Israel. The average number of years of working in the field was 13.64 (SD = 11.31), and most of the participants were medical oncologists and senior physicians (for a detailed description, see Table 1). We examined the associations between the demographic and medical variables (that had variability) and study variables through Pearson correlations, *t*-tests, and ANOVAs. No significant associations were found, except a significant difference between female oncologists and male oncologists in levels of reported burden t(70) = −2.36, *p* < 0.05). Female oncologists reported higher levels of burden (27.74, SD = 5.57), compared to male oncologists (24.27. SD = 6.87).

As can be seen from Table 2, the oncologists reported high levels of STS and burnout (17.29 and 26.44, respectively). These scores were around the cutoffs that were set by Stamm [16] for the identification of high levels of compassion fatigue (17 for STS and 27 for burnout). The oncologists reported moderate levels of compassion satisfaction; that is, the average score of the sample was in accordance with the mid-score reported by Stamm [16]. 

The oncologists reported high levels of internal LOC, low levels of external LOC/powerful others, and low levels of external LOC/chance. In addition, they reported moderate levels of guilt and low levels of helplessness. It should be noted that the meaning of these scores was established relative to the absolute middle of the scales.

### The Mediation Hypotheses

An analysis of the mediating role of guilt and helplessness in the association between oncologists’ LOC and their compassion fatigue (i.e., STS and burnout) and compassion satisfaction was conducted via SEM. An examination of the regression weights revealed insignificant associations between external LOC/chance, burnout, and the other study’s variables; as such, it was decided to exclude external LOC/chance and burnout from the final study’s model. As none of the demographic variables were associated with those study variables that were entered into the final model, these were not included in the analyses.

**Figure 1:**
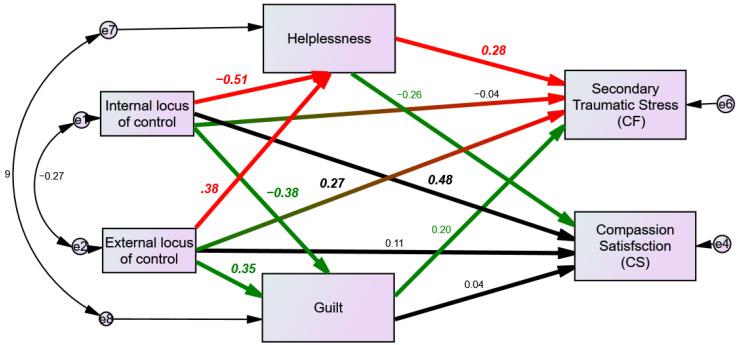
The mediation hypotheses (red and green paths) via SEM analysis. Bold numbers representing significant associations.

The overall fit of this final model was acceptable, χ^2^(1) = 0.048, *p* = 0.83; CFI = 1; TLI = 1.09, RMSEA = 0.00. The mediating hypotheses were tested, and a significant indirect effect was found between external LOC and STS, with helplessness and guilt mediating this association (standardized indirect effect = 0.18, *p* = 0.001). In addition, a significant indirect effect was found between internal LOC and STS, with helplessness and guilt mediating this association (standardized indirect effect = −0.22, *p* = 0.002). No mediating effects were found between oncologists’ LOC and compassion satisfaction. The model, with the standardized regression coefficients and their significance level, is depicted in Figure 1.

As can be seen from Figure 1 and Table 3, the hypothesis about a positive association between oncologists’ external LOC (others) and secondary traumatic stress (one aspect of compassion fatigue) is supported by our findings. A direct effect was found between oncologists’ external LOC (others) and secondary traumatic stress (one aspect of compassion fatigue). In addition, the mediating role of helplessness in this association was supported by an indirect effect. In other words, oncologists characterized by high levels of external LOC (others) reported higher levels of secondary traumatic stress (one aspect of compassion fatigue). Moreover, they reported higher levels of helplessness, which were associated with higher levels of secondary traumatic stress (one aspect of compassion fatigue).

The hypothesis about the associations between oncologists’ internal LOC and compassion fatigue was supported by our findings, only through the indirect effect, when helplessness mediated the association between oncologists’ internal LOC and oncologists’ secondary traumatic stress (one aspect of compassion fatigue). In addition, there was a positive direct association between oncologists’ internal LOC and compassion satisfaction. In other words, oncologists characterized by high levels of internal LOC reported lower levels of helplessness, and therefore lower levels of secondary traumatic stress (one aspect of compassion fatigue). In addition, they reported higher levels of compassion satisfaction.

The mediating role of guilt in the association between LOC and oncologists’ compassion fatigue and satisfaction was not supported by the study’s results. However, guilt was negatively associated with oncologists’ internal LOC and positively associated with oncologists’ external LOC.

## 4. Discussion

In the current study, we examined the association between oncologists’ LOC and compassion fatigue and satisfaction, and suggested two possible mediating variables: guilt and helplessness. The high levels of compassion fatigue (both secondary traumatic stress and burnout) that oncologists reported in the current study, as in previous studies [5,6,7,8,9,10,11,12,13,14,15], which might hinder the quality of care they provide to patients as well as their own personal well-being [7], highlight the importance of observing risk as well as resilience factors. The study’s results emphasize the significance of LOC as a predisposition that might, on the one hand, put oncologists at risk of experiencing compassion fatigue and burdensome feelings, such as helplessness and guilt. Alternatively, LOC may serve as a protective factor against feelings of helplessness and guilt and as a resilience factor associated with compassion satisfaction. Our results are in accordance with the results from the study by Ramondetta et al., which found that, among 273 gynecologic oncologists, external LOC was positively associated with work-related stress, whereas internal LOC was negatively associated with work-related stress [21].

Oncologists who hold the perception that they have no influence or control over events and reality (external LOC) seem to be vulnerable to feelings of helplessness, and therefore to the experience of STS in response to their work with suffering and dying patients. The experience of helplessness and lack of control in this context is quite similar to the role played by helplessness and lack of control in the development of post-traumatic stress disorder (PTSD) [32,33,34]. Criterion A of the DSM-5 [35] in the diagnosis of PTSD includes: “Indirect exposure to aversive details of the trauma, usually in the course of professional duties.” Oncologists are daily exposed directly and indirectly to multiple traumas via their patients’ suffering and deaths, and when they lack a sense of personal control, they may experience secondary traumatic stress.

However, it is not just the perception (i.e., the cognitive aspect), but also the feeling of helplessness that seems to contribute to the experience of secondary traumatic stress. Specifically, in the current study, the emotional component was found to mediate the association between external LOC (i.e., the cognitive perception) and secondary traumatic stress. Figley [36] termed this phenomenon “the cost of caring”, the emotional experience of helplessness, which seems to find expression in secondary traumatic stress.

In the current study, the oncologists who held perceptions of personal control (i.e., internal LOC) reported lower levels of secondary traumatic stress and higher levels of compassion satisfaction. As such, internal LOC seems to have acted as a protective factor against compassion fatigue, but more than that, it seems to have contributed to a sense of fulfillment and satisfaction. In other words, the perception of personal control seems to have resulted in a positive emotional state and positive feelings derived from the oncologists’ medical practice, such as the pleasure from and sense of fulfillment and success in helping their patients. It is possible that perceptions of personal control even in the face of death enable oncologists to avoid being overwhelmed, to maintain close, empathic, and compassionate relationships with their patients, and to have positive feelings regarding their work. This ability may be reflected in the shift toward a palliative approach in the care of cancer patients. In accordance with this approach, caring is a central feature of, and even the purpose of oncologists’ work, certainly when a cure is not possible [37]. It may be that case that taking care of patients and trying to alleviate their suffering provides oncologists meaning in the face of a patient’s death.

In the current study, guilt, although associated with LOC, was not found to mediate the association between LOC and compassion fatigue or satisfaction. Moreover, its associations with LOC ran contrary to the hypothesis. Namely, guilt was positively associated with external LOC and negatively associated with internal LOC. This finding raises a question as to whether the perception of personal responsibility is an outcome of the perception of personal control. In the current study, paradoxically, the oncologists who perceived themselves as having more of an ability to influence reality felt less guilt, and the oncologists who perceived themselves as having less of an ability to influence and control reality felt more guilt. As such, it is reasonable to suggest that guilt may operate as a mechanism intended to preserve the feeling of personal control [33,34]. In other words, it may be that oncologists who have an external LOC, who do not hold an internal working model of personal control over events, adopt guilt as a mechanism that enables them to maintain some personal control in the face of an uncontrollable reality of suffering and death. On the other hand, oncologists who have an internal LOC may not need guilt in order to face an uncontrollable situation and reality, as their internal working model of personal control may provide them with a firm enough basis to engage in this kind of work.

The current study had a few limitations. First, its cross-sectional nature does not allow for causal inferences to be made, nor did it allow us to trace changes over time. Second, the study was based on a relatively small sample, although it is important to note that the sample represents almost half of the oncologist population in Israel.

## 5. Conclusions

The current study demonstrates that oncologists are a population at risk of experiencing compassion fatigue—burnout and secondary traumatic stress—and views oncologists’ LOC as a predisposition associated with this phenomenon, stressing the cognitive as well as the emotional aspects of control as important factors associated with compassion fatigue, specifically with secondary traumatic stress. Whereas a lack of perception of personal control seems to put oncologists at risk of secondary traumatic stress, the perception of a sense of personal control seems to protect them. Furthermore, the perception of personal control was also found to be associated with compassion satisfaction: a sense of meaning, fulfillment, and pleasure. In summary, interventions that focus on compassion fatigue among oncologists should take into account not just the situational characteristics of the oncology setting, but also oncologists’ personality characteristics, such as LOC.

## Figures and Tables

**Table 1:** Demographic and medical variables by oncologists.

Variable	N (%)
Country of birth	
Israel	50 (68.5)
Other	23 (31.5)
Marital status	
Married or living with partner	70 (95.9)
Single	2 (2.7)
Divorced	1 (1.4)
Children	
Yes	61 (83.6)
No	10 (13.7)
Missing	2 (2.7)
Career status	
Intern	23 (31.5)
Fellow	49 (67.1)
Missing	1 (1.4)
Oncology specialty	
Clinical oncologist	58 (79.5)
Radiation oncologist	14 (19.2)
Missing	1 (1.4)
Number of work hours	
Part-time	6 (8.2)
Full-time	66 (90.4)
Missing	1 (1.4)
Primary practice setting	
Hospital	68 (93.2)
Community practice	3 (4.1)
Other	2 (2.8)
Medical specialty	
Breast oncologist	17 (23.3)
Thoracic oncologist	7 (9.6)
Hematologist oncologist	14 (19.2)
Other	26(35.6)
No medical specialty	9 (12.3)
Training/practice in palliative care (82)	
Yes	43 (58.9)
No	30 (41.1)

**Table 2:** The average scores and SDs of the study’s variables.

	Secondary Traumatic Stress	Burnout	Compassion Satisfaction	Internal LOC	External LOC—Others	External LOC—Coincidence	Learned Helplessness	Situational Guilt
Mean	17.29	26.44	36.83	23.10	10.88	10.92	1.90	2.79
S.D.	7.35	6.42	6.98	3.91	4.36	4.02	0.35	0.73

**Table 3:** Standardized regression weights.

	Beta	Sig.
External locus of control—others → Secondary traumatic stress (STS)	0.27	*
External locus of control—others → Learned helplessness	0.38	***
External locus of control—others → Situational guilt	0.35	***
Internal locus of control → Compassion satisfaction (CS)	0.48	***
Internal locus of control → Learned helplessness	−0.51	***
Internal locus of control → Situational guilt	−0.38	***
Learned helplessness → Secondary traumatic stress (STS)	0.28	*p* = 0.05

* *p* ≤.005, *** *p* ≤ 0.01.

## Data Availability

The data that support the findings of this study are available from the corresponding author upon reasonable request.
